# Carbonate Hydroxyapatite and Silicon-Substituted Carbonate Hydroxyapatite: Synthesis, Mechanical Properties, and Solubility Evaluations

**DOI:** 10.1155/2014/969876

**Published:** 2014-03-02

**Authors:** L. T. Bang, B. D. Long, R. Othman

**Affiliations:** ^1^Rekagraf Laboratory, School of Materials and Mineral Resources Engineering, Universiti Sains Malaysia, 14300 Nibong Tebal, Malaysia; ^2^Department of Mechanical Engineering, Faculty of Engineering, University of Malaya, 50603 Kuala Lumpur, Malaysia

## Abstract

The present study investigates the chemical composition, solubility, and physical and mechanical properties of carbonate hydroxyapatite (CO_3_Ap) and silicon-substituted carbonate hydroxyapatite (Si-CO_3_Ap) which have been prepared by a simple precipitation method. X-ray diffraction (XRD), Fourier transform infrared spectroscopy (FTIR), X-ray fluorescence (XRF) spectroscopy, and inductively coupled plasma (ICP) techniques were used to characterize the formation of CO_3_Ap and Si-CO_3_Ap. The results revealed that the silicate (SiO_4_^4−^) and carbonate (CO_3_^2−^) ions competed to occupy the phosphate (PO_4_^3−^) site and also entered simultaneously into the hydroxyapatite structure. The Si-substituted CO_3_Ap reduced the powder crystallinity and promoted ion release which resulted in a better solubility compared to that of Si-free CO_3_Ap. The mean particle size of Si-CO_3_Ap was much finer than that of CO_3_Ap. At 750°C heat-treatment temperature, the diametral tensile strengths (DTS) of Si-CO_3_Ap and CO_3_Ap were about 10.8 ± 0.3 and 11.8 ± 0.4 MPa, respectively.

## 1. Introduction

The use of hydroxyapatite (HA) as bone substitute is well known for its bioactivity and osteoconductivity *in vivo* [1, 2]. However, the natural bone which differs from pure HA contains about 4–8 wt% carbonate along with several multisubstituted ions (Na^+^, Mg^2+^, K^+^, F^−^, Cl^−^, etc.) in its structure [3–5]. Carbonate substituted into the HA structure (CO_3_Ap) is of special interest because the CO_3_^2−^ ion has an impact on different pathologies of human tissues, such as dental caries [6]. CO_3_Ap was also reported to be more soluble *in vivo* than HA and to increase the local concentration of calcium and phosphate ions that are necessary for new bone formation [7]. Moreover, CO_3_Ap is resorbed faster by osteoclasts and replaced with the new bone at a higher rate compared to HA [8]. CO_3_^2−^ ion can replace OH^−^ or PO_4_^3−^ ions giving A- or B-type CO_3_Ap, respectively. If these substitutions take place simultaneously, an AB-type substitution occurs, as in the case of the bone mineral [7, 9].

It was reported that Si enhances and stimulates osteoblast-like cell activity [10] *in vitro* and induces a higher dissolution rate *in vivo* [11]. The solubility was observed to increase with a decrease in structural order due to the presence of the foreign ions (i.e., CO_3_^2−^, SiO_4_^4−^) in the HA structure [12]; nonetheless, only few papers have investigated ion release in synthetic fluids [11, 13]. Therefore, the development of synthetic HA powders with a fully completed ionic substitution in the HA lattice is of great importance in order to mimic that of the natural bone.

Numerous research works have focused on the synthesis of HA biomaterial substituted with single- or multi-ion substitution of CO_3_^2−^ [14], Si^4+^ [3, 15], and so forth, whereas the substitution of CO_3_^2−^ along with other cations in the apatite structure was restricted to the cosubstitution of HA with the ionic pair of Mg^2+^/CO_3_^2−^ [4, 16], Sr^2+^/CO_3_^2−^ [17], and Na^+^/CO_3_^2−^ [18]. Although a few research works have been carried out on the synthesis of SiO_4_^4−^/CO_3_^2−^ cosubstitution in HA [13, 19], it is not clearly apparent whether SiO_4_^4−^ present in the material substituted completely the PO_4_^3−^ in the HA structure or whether the replacement was partial. It was reported [12] that both CO_3_^2−^ and SiO_4_^4−^ reduced HA crystallinity, and the structure could host only a limited amount of the two ions before collapsing. Additionally, the final product contained CO_3_^2−^ and SiO_4_^4−^, but there was a lack of experimental evidence on the competitive substitution of CO_3_^2−^ and SiO_4_^4−^ ions for PO_4_^3−^ ions [12]. Recently, an extensive study on the SiO_4_^4−^ and CO_3_^2−^ cosubstituted HA was reported [18]. However, the preparation methods were carried out under air atmosphere and used CO_2_ from the atmosphere as the CO_3_^2−^ source, and as such, there was no control of CO_3_^2−^ substitution level. Thus, the CO_3_^2−^ ion present could indeed be doped-HA, where the foreign ion is just adsorbed on the surface of the crystals [12]. Moreover, there were few research works that studied the mechanical properties of the ion-substituted HA after heat-treatment.

Therefore, the purpose of the present work is to investigate the simultaneous substitution of SiO_4_^4−^ and CO_3_^2−^ into the HA structure in order to obtain a product which is closer to the natural bone. The competition between CO_3_^2−^ and SiO_4_^4−^ for substituting the PO_4_^3−^ ions in the HA structure was also investigated. The aim of the work was also to evaluate the mechanical properties and the solubility of the silicon-substituted carbonate HA as compared to that of carbonate HA.

## 2. Experimental Procedure

A precipitation method was adopted to prepare CO_3_Ap using Ca(OH)_2_ (96% purity, FLUKA, 21181) and H_3_PO_4_ (15M, MERCK, 100573, Germany) with CO_2_ gas as the carbonate source [14]. The Ca/P molar ratio of the precursors was designed to be similar to Ca/P molar ratio of biological bone, which is 1.67 [2]. Initially, a solution of 300 mL of H_3_PO_4_ 1 M was gradually added to 500 mL of Ca(OH)_2_ 1 M under vigorous stirring at 400 rpm, whilst CO_2_ gas was passed through the reaction flask during the reaction. According to Landi et al. [14], to obtain the highest carbonation degree and favor B-type CO_3_Ap precipitation with respect to A-type, the CO_2_ flow was set at 0.5 bubble/s as the outlet flux. Similar to CO_3_Ap, the Si-CO_3_Ap was prepared using silicon tetra-acetate [Si(COOCH_3_)_4_] (98% purity, SIGMA-ALDRICH) as the Si precursor. Based on the chemical formula proposed by Gibson et al. [20] for silicon-substituted HA (Si-HA), the amount of reagents was calculated by assuming that one SiO_4_^4−^ ion would substitute for one PO_4_^3−^ ion based on a stoichiometric HA; Ca/(P+Si) molar ratio = 1.67. Si(COOCH_3_)_4_ was dissolved in the Ca(OH)_2_ solution under continuous stirring for 2 hours before adding the H_3_PO_4_ solution. In this research work, the Si content was chosen to be 1.6 wt% which had been shown to be the optimum amount for the enhancement of the mechanical properties of Si-HA reported in our previous study [21], where the Ca/(P+Si) ratio = 1.84.

The reactions took place in a reaction flask which was placed in a heating mantle to control the reaction temperature at 40°C ± 1. The pH of the solution was monitored using a pH meter. NH_4_OH 29% (J.T.Baker, USA) was added to maintain the pH of the solution at 9.4 ± 0.1. After the reaction was completed, the slurry was continuously stirred for 2 h without CO_2_ gas. It was then allowed to mature at room temperature for 24 h. Subsequently, it was filtered and washed with deionized water to remove any residue before being dried in an oven at 70°C for 24 h. The dried CO_3_Ap and Si-CO_3_Ap powders were then ground with an agate pestle and mortar. For the DTS test, the CO_3_Ap and Si-CO_3_Ap powders were compacted by uniaxial hydraulic pressing equipment using a die with 8 mm diameter at a pressure of 10 MPa. The thickness of samples was about 2.91–3.25 cm. Alcohol 70% was used to clean the mold. The compacted samples were then heat-treated at different temperatures of 650, 700, and 750°C with a heating rate of 3°C/min and soaked for 2 h in CO_2_ atmosphere (80 mL/min) which was passed through 150 mL distilled water. The syntheses of CO_3_Ap and Si-CO_3_Ap were repeated three times to confirm the reproducibility of the materials.

The as-synthesized and heat-treated powders were characterized using an X-ray diffractometer (XRD; D5000 Siemens) for phase identifications. Peak (002) was chosen for determining the crystallite size since it is one of the strongest peaks without any overlapping in the CO_3_Ap and Si-CO_3_Ap patterns. The lattice parameters (*a* and *c*) of the as-prepared CO_3_Ap and Si-CO_3_Ap samples were determined through the (*hkl*) peaks position of the apatite from XRD patterns according to (1) as follows [22, 23]:

(1) $$\begin{array}{c} \frac{1}{d^{2}}=\frac{4}{3}\left(\frac{h^{2}+kh+l^{2}}{a^{2}}\right)+\frac{l^{2}}{c^{2}}. \end{array}$$

Fourier transform infrared spectroscopy (FTIR; Perkin-Elmer FT-IR 2000, FTIR spectrometer) was used to study the silicon and carbonate substitutions of the different functional groups, such as OH^−^, PO_4_^3−^, CO_3_^2−^, and SiO_4_^4−^ in the CO_3_Ap and Si-CO_3_Ap samples. The carbonate content of powders was analyzed using an elemental analyzer (CHN test; Perkin Elmer series 2, 2400 CHNS/O). The chemical composition (Si and Ca) was determined by inductive coupled plasma (ICP) spectrometer (ICP/AES, ARL-3410). X-ray fluorescence spectrometer (XRF; Rigaku RIX-300 wavelength dispersive) was used to study the Ca/P ratio of the as-prepared powders. The particle size of the powder (with ultrasonic dispersion) was measured using a Malvern Mastersizer X (Malvern Instruments, Malvern, UK). The powder before being characterized had been passed through a 75 *μ*m sieve.

The densities of the heat-treated CO_3_Ap and Si-CO_3_Ap compacts were measured using Archimedes' principle. The diametral tensile strengths (DTS) of the heat-treated CO_3_Ap and Si-CO_3_Ap compacts were tested at a strain rate of 0.5 mm/min. The DTS test involves compressing a sample diametrically, inducing a stress that causes the sample to yield in tension. In this test, a disk sample was placed between two platens and then vertically compressed until it broke [24]. During loading, the applied force was recorded and the tensile stress was calculated using (2)

(2) $$\begin{array}{c} F_{t}=\frac{2P_{\max}}{\pi dh}, \end{array}$$

where *P*_max⁡_ is maximum load at failure (N) and *h* and *d* are the thickness and diameter of the compacts (mm), respectively. The solubility evaluation was performed in triplicate on the as-synthesized CO_3_Ap and Si-CO_3_Ap compacts (8 mm diameter die, 10 MPa) by immersing the compacts in a simulated body luid (SBF) solution at 36.5°C. The SBF solution was prepared according to the procedure described by Kokubo and Takadama [25]. The tests were carried out within 1 and 7 days. After the predetermined soaking time, the samples were removed and the liquid mediums were analyzed by ICP. The released ion was estimated by subtracting the initial ion concentration of the SBF solution from the ion concentration of the SBF solution after immersion.

Statistical analysis was performed to evaluate the statistical differences between the sample sets by employing one factor analysis of variance (ANOVA) when comparing more than two sample populations. Significant differences were considered at the 95% level (*P* < 0.05).

## 3. Results and Discussion

### 3.1. Physical and Chemical Composition Analyses

Table 1 shows the physical and chemical properties of the as-synthesized CO_3_Ap and Si-CO_3_Ap samples. The mean particle size of the as-synthesized Si-CO_3_Ap sample is significantly smaller than that of the as-synthesized CO_3_Ap sample. This can be attributed to the substitution of Si in the HA structure, as reported in previous research works [21, 26].

In the same table, the Ca/P molar ratios of the as-synthesized CO_3_Ap and Si-CO_3_Ap samples show much higher values than those of the predetermined ratios. This indicated that the substitution of CO_3_^2−^ and SiO_4_^4−^ ions for the PO_4_^3−^ groups in the HA had taken place. These substitutions reduce the amount of PO_4_^3−^ group, thus leading to an increase in the Ca/P ratio [14, 20]. However, the Ca/P ratio in this study was in the range of the Ca/P molar ratio of CO_3_Ap reported previously, which was of 1.7–2.6 [27].

The Si contents are also included in Table 1. Si measured in the as-synthesized Si-CO_3_Ap sample is about 0.85 wt%, and this is much lower than the starting value (1.6 wt%). The rest of the Si unaccounted for will be explained in the FTIR analysis. It was suggested that an amount of only 1 wt% Si substituted into HA was sufficient to elicit important bioactive improvements [12], and, hence, the Si-substituted CO_3_Ap in this research work could be considered to approach this enhancement.

After heat-treatment at a temperature range of 650–750°C, the carbonate amount slightly decreases compared to the as-prepared samples (Table 2). This is due to the fact that carbonate absorbed had desorbed upon heat-treatment. The amount of carbonate is close to the typical amount of carbonate in human bone [28].

### 3.2. XRD Analysis

**Figure 1:**
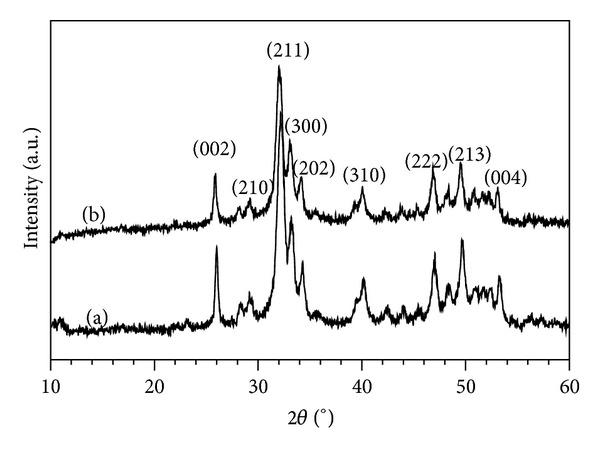
XRD patterns of the as-prepared powders: (a) CO_3_Ap and (b) Si-CO_3_Ap.

Figure 1 shows the XRD patterns of the as-synthesized CO_3_Ap and Si-CO_3_Ap powders. The broad peaks indicate the formation of HA phase with low crystallinity, and no secondary crystalline phases were observed. The poor crystallinity was due to the low synthesis temperature and the substitution of SiO_4_^4−^ and CO_3_^2−^ ions limited the crystallization of the HA phase [18, 21].

The crystallite size determined using Scherrer's equation and the lattice parameters are given in Table 3. The CO_3_^2−^ and SiO_4_^4−^ substitutions in HA structure led to changes in the crystal lattice parameters [4, 18]. Previous studies had shown that the *a*-axis decreased and the *c*-axis increased with increasing CO_3_^2−^ or SiO_4_^4−^ in the HA structure [3, 6]. The values presented in Table 3 for the as-prepared powders in this present research work also show a similar trend with previous works. The SiO_4_^4−^ groups are larger and have a more negative charge than either PO_4_^3−^ or CO_3_^2−^ ions [15, 18]. Additionally, the substitution of SiO_4_^4−^ and CO_3_^2−^ for PO_4_^3−^ contributes to reducing the crystallite size, as has been observed previously in other studies [12, 18, 21].

Numerous studies showed that both *a*- and *c*-axis dimensions increased with the silicon content [18, 29, 30]. Considering the substitution of SiO_4_^4−^ in the CO_3_Ap, it is possible that, *a*- and *c*-axis dimensions are higher than those of CO_3_Ap (Table 3) because the ionic bond length of a Si–O bond (0.166 nm) is greater than that of P–O bond (0.157 nm). The radius of the PO_4_^3−^ tetrahedron would be smaller than that of the SiO_4_^4−^ tetrahedron that results in the change of HA lattice parameters.

**Figure 2:**
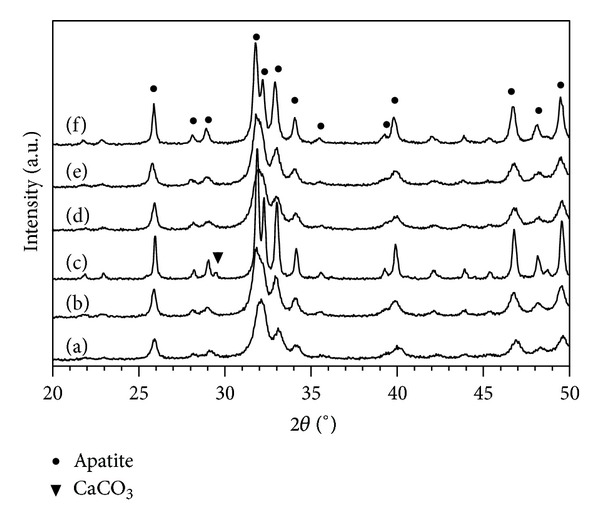
XRD patterns of the samples after heat-treatment of Si-CO_3_Ap at (a) 650°C, (b) 700°C, and (c) 750°C and of CO_3_Ap at (d) 650°C, (e) 700°C, and (f) 750°C.

After heat-treatment at 650°C to 700°C, pure CO_3_Ap and Si-CO_3_Ap are still observed and no secondary phases are detected (Figure 2(a), (b), (d), and (e)). However, a new phase, CaCO_3_, is clearly observed in Si-CO_3_Ap samples heat-treated at 750°C due to the decomposition of the Si-CO_3_Ap samples.

Sintering of CO_3_Ap at high temperatures (≥900°C) [15, 31] produces hydroxyapatite (HA) and CaO. In the CO_2_-rich atmosphere, the CaCO_3_ obtained was due to the reaction of CaO and CO_2_. Therefore, a mixture of CO_3_Ap and CaCO_3_ is observed after heat-treatment in CO_2_ atmosphere. The decomposition temperature decreased with an increase of the carbonate [31] and/or silicon content [15, 32]. Since the heat-treatment process was carried out at low temperatures, such decomposition did not occur in the CO_3_Ap sample but did occur in Si-CO_3_Ap sample at 750°C. The simultaneous substitution of SiO_4_^4−^ and CO_3_^2−^ ions for the PO_4_^3−^ ions of the HA structure increased the defects in HA structure and produced more OH^−^ vacancies [13] compared to CO_3_Ap. The formation of OH vacancies has been proven to accelerate the decomposition process [23]. Thus, the formation of CaCO_3_ in the Si-CO_3_Ap could be explained by a similar mechanism as the decomposition of CO_3_Ap.

### 3.3. FTIR Analysis

**Figure 3:**
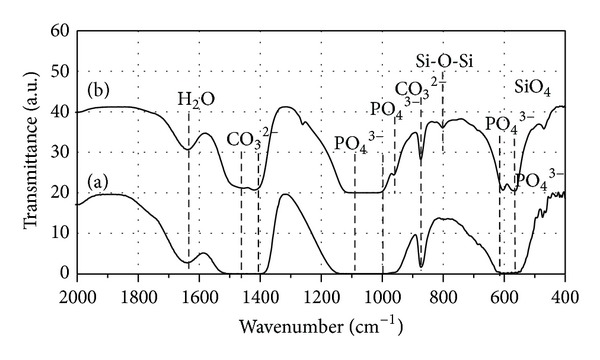
FTIR spectra of the as-prepared powders: (a) CO_3_Ap and (b) Si-CO_3_Ap.

FTIR spectrum of each powder (Figure 3) shows the characteristic absorption bands of HA corresponding to stretching vibration of PO_4_^3−^ ions at 567, 604 cm^−1^ (*υ*4); 963 cm^−1^ (*υ*1); 1045 cm^−1^ (*υ*3); in all the as-synthesized powder bands. The broad band at about 1638 cm^−1^ corresponds to in-plane water bending mode. The CO_3_^2−^ groups substituted in B-site were confirmed with typical bands around 874 cm^−1^ (*υ*2), 1470 cm^−1^ [4, 18, 33], whereas the bands located at 1505 cm^−1^ could be attributed to A-type CO_3_Ap [28].

The characteristic OH^−^ bands of HA at 630 cm^−1^ are not clearly visible in all FITR spectra. In fact, a similar decrease in the intensity of OH^−^ signals was also observed due to the substitution of CO_3_^2−^ at the OH^−^ lattice of HA [33]. In this case, the substitution of CO_3_^2−^ and SiO_4_^4−^ ions for PO_4_^3−^ would create an OH^−^ loss needed to compensate the charge balance, thus resulting in the weak of OH^−^ signal.

Additional bands are also observed in the Si-CO_3_Ap sample at about 800 cm^−1^ and 480 cm^−1^ which do not appear in CO_3_Ap sample. The band at 480 cm^−1^ is assigned to the SiO_4_^4−^ in the apatite structure [15]. However, the band at about 800 cm^−1^ might be assigned to either the silicate group [30] or to the O–Si–O bending in the SiO_2_ amorphous phase [22, 34]. As detected by ICP, the amount of Si in Si-CO_3_Ap sample is much lower than the starting value (Table 1); the silicate species which could not totally be incorporated in the apatite structure exist on the surface of the materials as an amorphous phase [22, 35] and/or remain in mother liquors after precipitation [36]. The remaining Si suggests that the competition arising between the SiO_4_^4−^ and CO_3_^2−^ ions occupies the PO_4_^3−^ sites. The polymerization of the silicate species at the surface was reported elsewhere [37]. In another research work [38], the amorphous SiO_2_ phase in *β*-TCP containing Si-substitution showed a significantly higher MC3T3-E1 osteoblast-like cell number compared to pure *β*-TCP. Therefore, the presence of SiO_2_ would not cause toxicity to the cells and would not affect cell differentiation.

The substitutions of CO_3_^2−^ and SiO_4_^4−^ groups for PO_4_^3−^ change the symmetry and stability of an apatite structure [39]. As a result of these substitutions, shifts and splitting of the PO_4_ vibration bands at about 500–700 cm^−1^ occur in the apatite IR spectra (Figure 3).

It has already been reported that the calcium phosphate apatite constituent of bone mineral consists of a mixed AB-type substitution [40]. The results from the present study confirm the formation of AB-type carbonated apatite along with the presence of Si in the structure. Thus, this complex substitution type is also of utmost importance when the development of a synthetic bone-substitute material is sought.

### 3.4. Evaluation of Mechanical Properties and Microstructure

**Figure 4:**
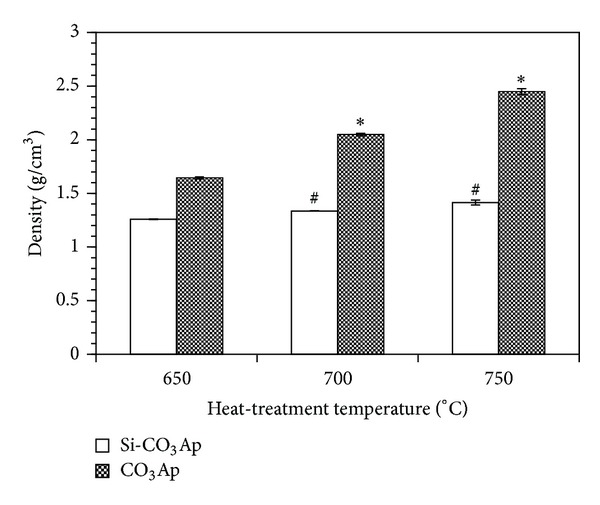
Density of samples after heat-treatment at different temperatures. *P < 0.05 and ^#^ *P* < 0.05, statistically different compared to CO_3_Ap and Si-CO_3_Ap heat-treated at 650°C, respectively; *n* = 8.

The mechanical and physical properties were evaluated in terms of diametral tensile strength (DTS) and bulk density. In Figure 4, the density of CO_3_Ap sample is higher than that of Si-CO_3_Ap sample at any heat-treatment temperatures. This can be explained by the higher lattice parameters of both CO_3_^2−^ and SiO_4_^4−^ cosubstitution compared to the single CO_3_^2−^ substitution (Table 3).

It can also be seen that the density of the CO_3_Ap samples significantly increases with increasing heat-treatment temperatures, whilst there is only a slight change in the density of the Si-CO_3_Ap samples. The substitution of Si reduced the density of the materials compared to HA as reported previously [15, 21] due to the change of unit cell parameters in the silicon-substituted materials. Therefore, the effect of silicon became significant which slowed down the densification process upon heat-treatment. In the present research work, the densities of CO_3_Ap and Si-CO_3_Ap are significantly lower compared to that of a fully dense HA (3.16 g/cm^3^) due to the low heat-treatment temperatures.

**Figure 5:**
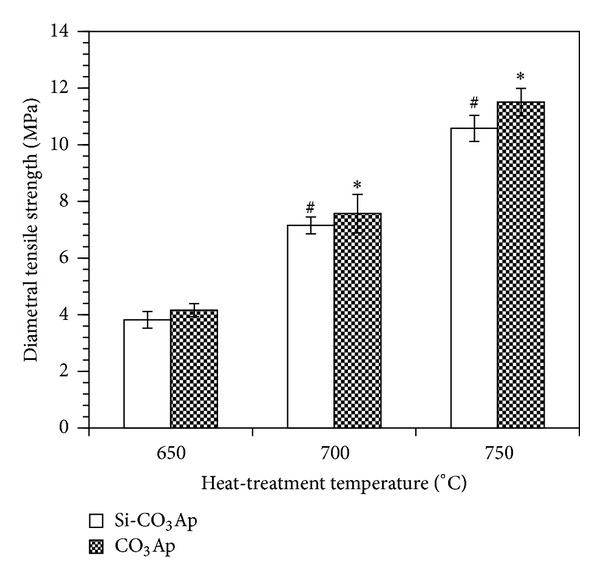
Diametral tensile strength (DTS) of samples at different temperatures. *P < 0.05 and ^#^ *P* < 0.05, statistically different compared to CO_3_Ap and Si-CO_3_Ap heat-treated at 650°C, respectively; *n* = 8.

Figure 5 shows that the DTS of both CO_3_Ap and Si-CO_3_Ap samples significantly increase with increasing temperatures. The increase in DTS value of CO_3_Ap with the increasing heat-treatment temperatures can be explained by the increase in density as shown in Figure 4. However, although a slightly higher density was obtained for the Si-CO_3_Ap, the DTS of Si-CO_3_Ap increases significantly with increasing heat-treatment temperatures. This is due to the cosubstitution of CO_3_^2−^ and SiO_4_^4−^. This cosubstitution induced the smaller particle size (Table 1). In addition, Si substitution was reported to impede grain growth during heat-treatment [41] and so increased the DTS value.

By comparison, the DTS of CO_3_Ap samples appear to be slightly higher compared to those of Si-CO_3_Ap samples. This difference in strength was evaluated to be *ρ* > 0.05, and as such, this difference in DTS value of Si-CO_3_Ap is insignificant compared to CO_3_Ap. However, its density is significantly lower (*ρ* < 0.05) indicating the positive effect of SiO_4_^4−^ and CO_3_^2−^ cosubstitutions on this matter. As reported, the effect of silicon on the increase of mechanical strength was evidenced at higher heat-treatment temperatures, that is, 1200°C and above, as compared to Si-free samples [21]. Conversely, at lower temperatures, this effect was not so apparent where the strengths of Si-samples and Si-free samples were comparable based on previous studies [21, 41] and even lower [19] due to the lower density. Hence, due to the low heat-treatment temperatures employed in this research work, the difference in strength between CO_3_Ap and Si-CO_3_Ap samples is not that significant.

**Figure 6:**
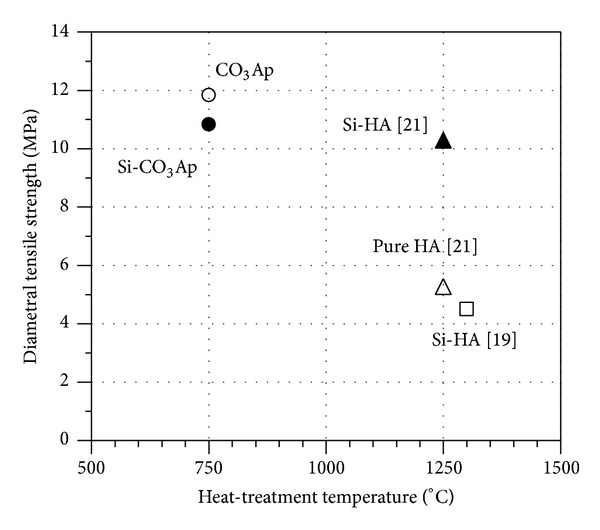
DTS versus heat-treatment temperatures for various carbonate hydroxyapatites.

Figure 6 presents a comparison of the DTS values of the present materials at 750°C with those of samples in previous research works [19, 21]. Interestingly, at the same Si content (about 0.8 wt%), the DTS values of CO_3_Ap and Si-CO_3_Ap at 750°C are about 10.8 ± 0.3–11.8 ± 0.4 MPa, and these are higher than those of Si-substituted HA samples at 1250°C [21] and much higher than that of Si-HA sample at 1300°C [19]. This demonstrates that, at these low heat-treatment temperatures, the cosubstitution of carbonate and Si in the HA structure would increase the strength of the final product.

In Figure 6, the DTS of Si-substituted HA sample [21] is higher than that of pure HA because the SiO_4_^4−^ substitution impeded grain growth at high temperatures and, therefore, increased the strength of the materials [41]. The DTS of CO_3_Ap and Si-CO_3_Ap in the present work are also higher than that of pure HA. It was explained [42] that the CO_3_^2−^ and SiO_4_^4−^ substitutions also reduced the grain size of the final product and resulted in an increase of the strength of the samples.

### 3.5. Solubility Evaluation

**Figure 7:**
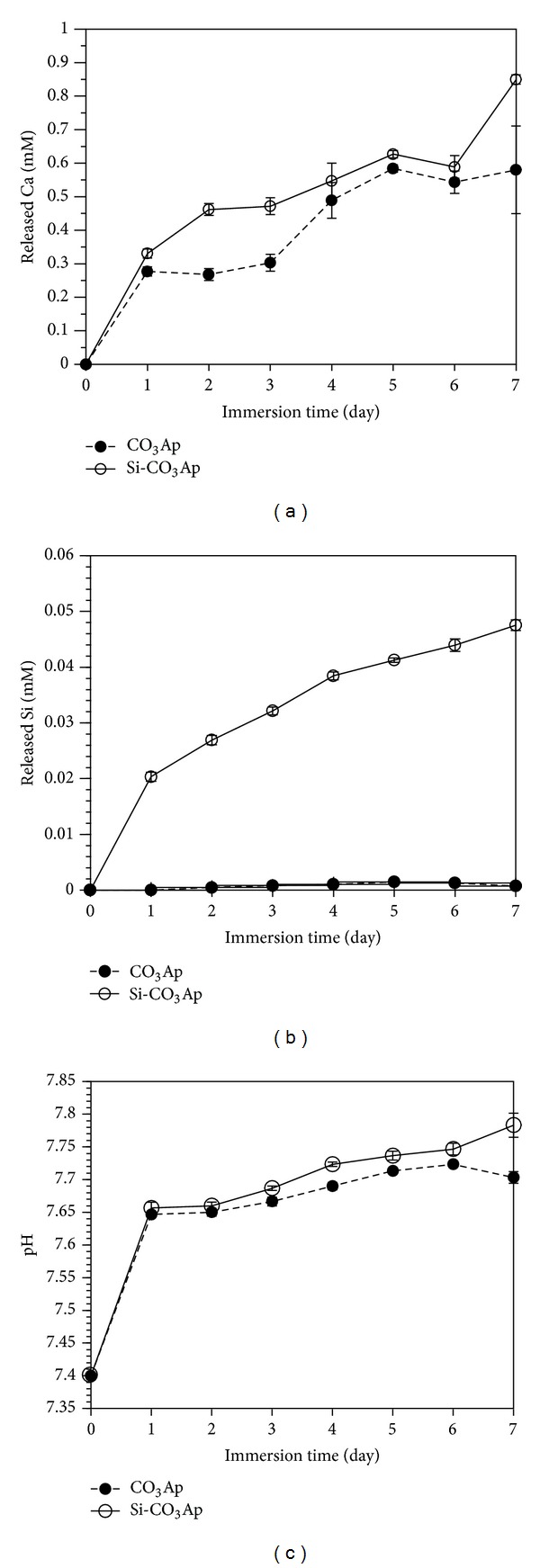
Released ions and pH of SBF solution after immersion: (a) released Ca, (b) released Si, and (c) pH.

In the case of crystalline HA, the degree of micro- and macroporosities, defect structure, and amount and type of other phases present have a significant influence on the dissolution rate [43]. In this study, the immersion of the CO_3_Ap and Si-CO_3_Ap compacts (surface area = 150.8 mm^2^) into SBF solution produced noticeable changes in the ion concentrations of the solution. Figures 7(a), 7(b), and 7(c) show the ion concentration of Ca, Si and changes in pH value of the medium after a certain period of immersion time, respectively. According to Boanini et al. [12], crystallinity and crystal dimensions significantly affected the solubility and, as a consequence, ion release. Thus, a decrease in structural order due to the presence of foreign ions might be responsible for the observed increase in solubility.

In Figure 7, the Ca^2+^ and Si^4+^ ion concentrations as well as the pH of the SBF solution increase with soaking duration which indicates the dissolution of Ca^2+^ and Si^4+^ ions. It had been reported that the initial dissolution of implant materials plays an important role in enhancing their bonding to the bone [32]. With an increase in the soaking duration, Ca^2+^ concentrations and pH value continuously increase due to the ionic exchange between H^+^ within the SBF solution and Ca^2+^ in the CO_3_Ap and Si-CO_3_Ap compacts [44, 45]. The increase of solution pH generally facilitates the nucleation of apatite [46].

The release of Si^4+^ ions was also observed continuously over the whole investigation period. It was reasoned out that the amorphous layer surrounding the apatite grains dissolved within the first period of immersion in SBF leaving a more stable and less soluble core [13]. As solubility is highly sensitive to the structural and chemical compositions of the apatite samples, the crystallite size is a key factor for *in vitro* behavior of synthetic apatite [47]. In this manner, the resorbability of CO_3_Ap and Si-CO_3_Ap could be promoted by a smaller crystallite size when CO_3_^2−^ and SiO_4_^4−^ were cosubstituted; the amorphous shell can be thicker and yield a more intense and prolonged ion release [13]. In addition, the Ca^2+^ release in Si-CO_3_Ap compacts is slightly higher compared to CO_3_Ap, which suggests a better solubility (Figure 7(a)) that leads to a faster super-saturation with respect to HA, a faster nucleation, and growth of apatite on the surface of the compacts [36].

By comparison, the Ca^2+^ release for CO_3_Ap and Si-CO_3_Ap samples in this study is much higher than that of Mg-substituted fluorapatite [48] and HA [44, 49] under the same conditions. It was reported that the solubility of materials increases with increasing ionic substitutions into the HA lattice and decreasing crystallinity which is represented by the higher ion release in the SBF solution [13, 16, 49]. Therefore, the CO_3_Ap and Si-CO_3_Ap obtained in this work are of higher solubility compared to the above-mentioned materials.

Based on the solubility evaluations using SBF, the solubility of CO_3_Ap and Si-CO_3_Ap is such that it is predicted that ions would continuously exist in actual physiological conditions. This is further reinforced by a previous work [13]. These materials could supply elements which are essential for osteoblast activity and new bone tissue formation [13]. The simultaneous presence of such elements can further enhance the cell response.

## 4. Conclusions

Carbonate hydroxyapatite and silicon-substituted carbonate hydroxyapatite powders were successfully synthesized by a simple and high-yield process. The crystallite and mean particle size of Si-CO_3_Ap sample was significantly smaller than that of CO_3_Ap sample due to the cosubstitution of SiO_4_^4−^ and CO_3_^2−^ in the HA structure. No secondary phases were detected in CO_3_Ap and Si-CO_3_Ap samples after heat-treatment in the temperature range of 650°C to 700°C. CaCO_3_ was observed in Si-CO_3_Ap sample after heat-treatment at 750°C, whilst the purity of CO_3_Ap was retained. The SiO_4_^4−^ and CO_3_^2−^ cosubstituted HA structure led to a significant decrease in density compared to a single CO_3_^2−^ substituted HA structure, whilst the DTS of both samples showed insignificant differences.

The competition between SiO_4_^4−^ and CO_3_^2−^ ions had taken place to occupy the PO_4_^3−^ site. Si-CO_3_Ap existed in the form of AB-type carbonated apatite, and the presence of SiO_4_^4−^ in the structure is of utmost interest in developing a synthetic bone-substitute material. The total amount of carbonate and silicon and the crystal size of the powder obtained mimic those of biological apatites. The silicon substitution improved the solubility of Si-CO_3_Ap which prolongs the ion release compared to that of Si-free CO_3_Ap. The present materials possess low crystallinity and the CO_3_^2−^ content is close to that found in natural bone, and, in combination with the high strength, these materials could be ideal for bone substitutes.

## Figures and Tables

**Table 1:** Physical and chemical properties of the as-synthesized CO_3_Ap and Si-CO_3_Ap samples.

Sample	Si content (wt%)		Ca/P		Mean particle size (*μ*m)
	Starting value	Measured value (ICP/in powder)	Starting value	Measured value (XRF)	
CO_3_Ap	0	—	1.67	2.08	2.52
Si-CO_3_Ap	1.6	0.85	1.84	2.16	0.98

**Table 2:** Carbonate contents in the CO_3_Ap and Si-CO_3_Ap samples before and after heat-treatment.

Sample	CO_3_ (wt%) As-prepared powders	CO_3_ (wt%) Heat-treated powders		
		650°C	700°C	750°C
CO_3_Ap	10.75	10.1	10.05	10.05
Si-CO_3_Ap	10.25	9.4	9.4	8.4

**Table 3:** Lattice parameters and crystallite size of the as-synthesized CO_3_Ap and Si-CO_3_Ap powders.

Sample	Lattice parameters (A°)		Crystallite size (nm)
	*a* ± 0.003	*c* ± 0.003	
HA [26]	9.4366	6.8905	—
CO_3_Ap	9.3860	6.8963	23.12 ± 0.03
Si-CO_3_Ap	9.4061	6.9057	16.82 ± 0.02
